# INC280, an orally available small molecule inhibitor of c-MET, reduces migration and adhesion in ovarian cancer cell models

**DOI:** 10.1038/srep11749

**Published:** 2015-07-03

**Authors:** Kim Moran-Jones, Laura M. Brown, Goli Samimi

**Affiliations:** 1 Kinghorn Cancer Centre and Garvan Institute of Medical Research, Darlinghurst, NSW, Australia; 2 St. Vincent’s Clinical School, Faculty of Medicine, UNSW Australia, NSW, Australia

## Abstract

5-year survival rates for ovarian cancer are approximately 40%, and for women diagnosed at late stage (the majority), just 27%. This indicates a dire need for new treatments to improve survival rates. Recent molecular characterization has greatly improved our understanding of the disease and allowed the identification of potential new targets. One such pathway of interest is the HGF/c-MET axis. Activation of the HGF/c-MET axis has been demonstrated in certain ovarian tumours, and been found to be associated with decreased overall survival, suggesting its potential as a therapeutic target. The objective of this study was to determine the efficacy of a novel, highly potent, orally-bioavailable c-MET inhibitor, INC280, in blocking cell phenotypes important in ovarian cancer metastasis. Using *in vitro* and *ex vivo* models, we demonstrate that INC280 inhibits HGF-induced c-MET, and reduces downstream signalling. HGF-stimulated chemotactic and random migration are decreased by INC280 treatment, to levels seen in non-stimulated cells. Additionally, HGF-induced adhesion of cancer cells to peritoneal tissue is significantly decreased by INC280 treatment. Overall, these data indicate that INC280 inhibits many cell behaviours that promote ovarian cancer metastasis, and merits further investigation as a therapeutic candidate in the treatment of patients with ovarian cancer.

Ovarian cancer is the 5^th^ leading cause of cancer-related deaths in women, and the deadliest of the gynaecological malignancies^1^. Epithelial ovarian cancer (EOC), which accounts for 90% of ovarian cancer diagnoses, can be classified either as Type I or II, with the latter being responsible for 70% of all EOC cases^2^. Overall 5-year survival rates for EOC have remained relatively stable over the past 2+ decades, at approximately 40 percent. In Type II cases, typically diagnosed at late stage (63%) when the disease has metastasized throughout the peritoneal cavity, the 5-year survival rate drops dramatically to only 27%. This has changed little since the introduction of platinum and taxane-based therapy (reviewed in^3^). Characterized by genomic instability^4^, the majority of these patients initially respond to chemotherapy, but present with chemo-resistant tumours within approximately 2 years, indicating a need for new strategies to treat ovarian cancer. While there are a number of agents under review for treatment of ovarian cancer (reviewed in^5^), studies and clinical trials are on-going to determine efficacy and relevant biomarkers.

One potential target that is currently under investigation is the hepatocyte growth factor (HGF)/c-MET signalling axis. HGF, the only known ligand of c-MET, is a paracrine factor, secreted predominantly by mesenchymal cells. Activation of c-MET promotes cell proliferation, survival, motility, and invasion, all features of tumour growth and progression (reviewed in^6^). During mammalian development, HGF is produced by mesenchyme within the uro-genital ridge, adjacent to c-MET-expressing epithelial cells, suggesting an involvement of this pathway in ovarian development and proliferation^7^. Over-expression of the c-MET receptor has been reported in a number of cancer types (reviewed in^6^). In ovarian cancer however, c-MET over-expression is not associated with c-MET mutation or gene amplification^8^, but may instead be secondary to mutations in other genes such as Ras and Ret^9^, or hypoxia^10^.

A number of studies have identified high expression of c-MET in subsets of all four of the major histotypes of EOC (high grade serous, clear cell, mucinous, and endometrioid^11^,^12^,^13^,^14^), and have demonstrated correlation with poorer prognosis^12^. Similarly, a high level of HGF in serum is an indicator of ovarian cancer in women presenting with a pelvic mass, and predictive of poor prognosis in women with advanced epithelial ovarian cancer^15^. In addition to being highly expressed in the reactive stroma of tumours^16^, HGF is also present at high levels in ovarian cancer ascites^17^. HGF can also induce up-regulation of the c-MET receptor^18^, setting in place an auto-amplification loop, and indicating the c-MET-HGF signalling pathway as a valuable target in EOC. A number of c-MET inhibitors and HGF antagonists are currently under investigation, in both pre-clinical models of ovarian cancer, and clinical trials for multiple cancer types (reviewed in^6^ and^19^). Most tyrosine kinase inhibitors (TKIs) against the c-MET receptor compete for the ATP-binding site in the tyrosine kinase domain, preventing trans-activation and recruitment of downstream effectors. Some are specific for c-MET, while others exhibit activity against several tyrosine kinase receptors (reviewed in^6^,^19^). Many of these agents have been tested in pre-clinical models of ovarian cancer including PF-2341066 (c-MET-specific), Foretinib (c-MET and VEGFR-2), MK8033 (c-MET specific), DCC-2701 (c-MET/Tie-2/VEGFR-2), and SU11274 (c-MET specific) and lead to decreased cell motility and invasion, reduced adhesion and peritoneal dissemination, as well as reductions in tumour burden in treated cells and animals^20^,^21^,^22^,^23^,^24^. A number of these TKIs are currently under clinical investigation for solid cancers.

Recently, Incyte/Novartis^25^ identified a novel c-MET inhibitor, INCB28060 (INC280). An ATP competitive inhibitor, INC280 is orally bio-available, demonstrates 10,000-fold selectivity for c-MET over a panel of human kinases, has an IC_50_ in the sub-nanomolar range, and remains at active concentrations in the plasma for several hours^25^. INC280 is currently in phase 1 trials (ClinicalTrials.gov Identifier: NCT01072266) as a therapeutic in multiple cancer types.

In this study, we investigate the effect of INC280 in a number of ovarian cancer cell models, and demonstrate decreases in activation of downstream signalling pathways, migration, and peritoneal adhesion.

## Results

### INC280 inhibits HGF-stimulation of c-MET phosphorylation, and downstream pathway activation

**Figure 1:**
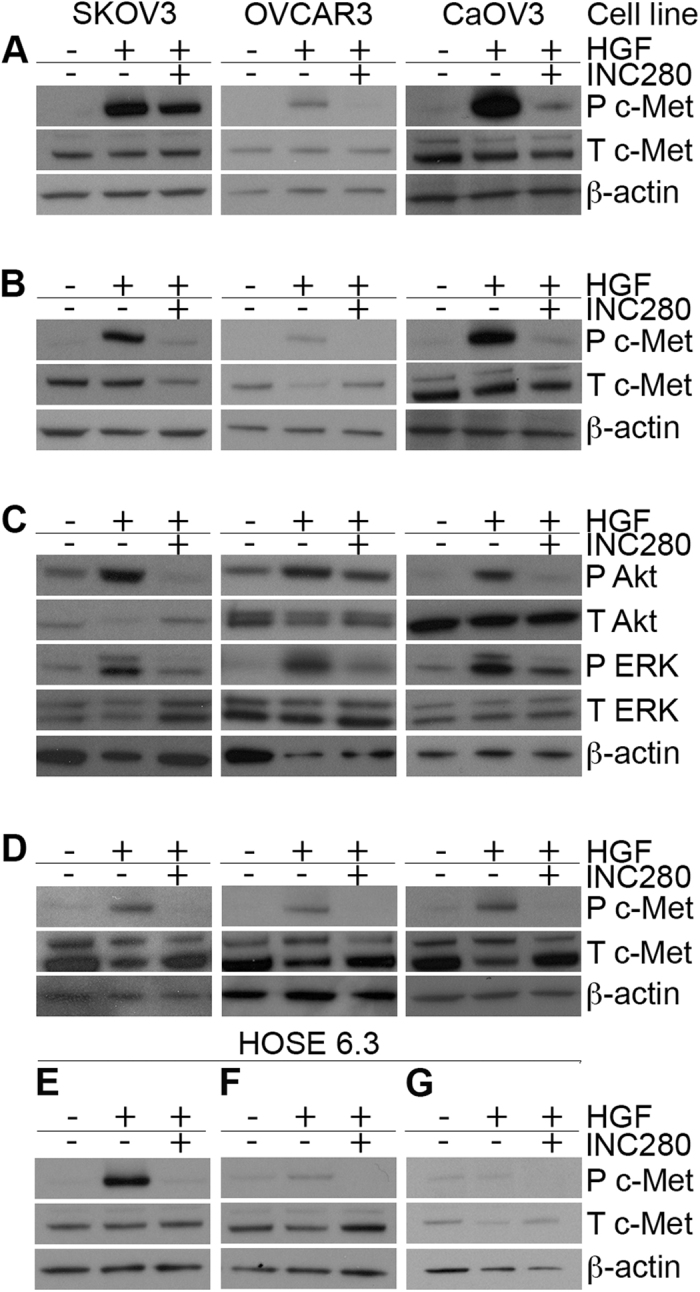
INC280 inhibits HGF-stimulated c-MET phosphorylation, and downstream signalling. 3 cell lines (SKOV3, OVCAR3, and CaOV3) were treated with HGF, HGF + INC280, or left untreated, for 10 min (**A**), 360 min (**B**), or 24 hrs (**D**). c-MET phosphorylation was assessed in these cell lines using antibodies against phosphorylated Y1234/1235 c-MET (P c-MET), and total c-MET (T c-MET). **C**) Signaling downstream of c-MET activation was assessed (shown here 360 minutes post treatment using antibodies against phosphorylated S473 AKT (P AKT), total AKT (T AKT), phosphorylated Y202/204 ERK 1/2 (P ERK), and total ERK. β-actin was used as a loading control. HOSE 6.3 normal ovarian surface epithelium cells were also treated with HGF, HGF + INC280, or left untreated, for 10 min (**E**), 360 min (**F**) or 24 hrs (**G**). Full length gels containing the cropped images above are shown in Supplementary Figures 4 (**A** and **B**), 5 (**C**), and 6 (**D–G**).

Similar to previous findings^13^,^23^, we demonstrate that the three ovarian cancer cell lines SKOV3, OVCAR3, and CaOV3, express low levels of phosphorylated c-MET under baseline conditions (Fig. 1A). c-MET phosphorylation is increased upon addition of 40 ng/mL HGF, whereas treatment with INC280 inhibits HGF-induced c-MET phosphorylation for at least 360 mins post-treatment (Fig. 1B). Similarly, while addition of HGF resulted in phosphorylation of AKT and ERK1/2, this activation was reduced upon treatment with INC280 (Fig. 1C), indicating inhibition of the c-MET downstream pathways PI3K and ERK/MAPK. HGF stimulation of c-MET phosphorylation persisted for up to 48 hrs, and examination of samples collected at 24 (Fig. 1D) and 48 hrs post-treatment indicated that INC280 decreased HGF-stimulated c-MET phosphorylation up to 48 hrs (Supplementary Figure 1). In HOSE 6.3 cells (immortalized normal human ovarian surface epithelial cells), baseline phospho-c-MET is low, but increases with addition of HGF (Fig. 1E–G), and is abrogated by INC280 treatment.

### INC280 reduces HGF-induced proliferation in vitro

**Figure 2:**
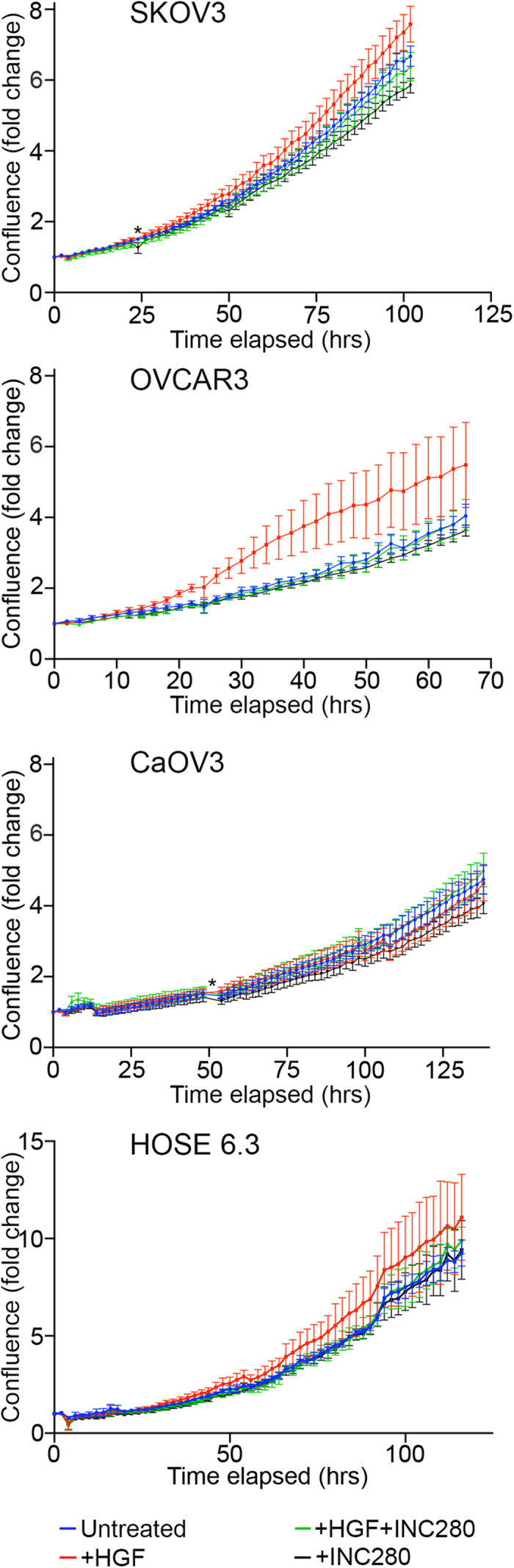
Proliferation of ovarian cancer cells in presence of HGF and INC280. Changes in the proliferation of SKOV3, OVCAR3, CaOV3, and HOSE 6.3 cell lines were measured (using confluence as a surrogate marker) in the presence of HGF and/or INC280. The composite curves of progressive fold-change in cell confluence relative to the end of serum starvation are plotted as means of 3 independent experiments with internal triplicates ± SEM. * a small number of time points were removed from analyses.

To investigate cell proliferation effects of c-MET inhibition by INC280, we tested the proliferation rates of untreated OVCAR3, SKOV3 and CaOV3 cancer cells, as well as the normal ovarian surface epithelium HOSE 6.3 cells, with HGF, HGF + INC280, and INC280 treatments (Fig. 2). Minor increases in proliferation were seen in OVCAR3, SKOV3, and HOSE 6.3 cells treated with HGF over 3–5 days post-treatment (not statistically significant), which were abrogated by addition of INC280. INC280 alone had no statistically significant effect on *in vitro* proliferation relative to untreated cells.

### INC280 inhibits HGF-stimulated chemotactic-induced and random migration

**Figure 3:**
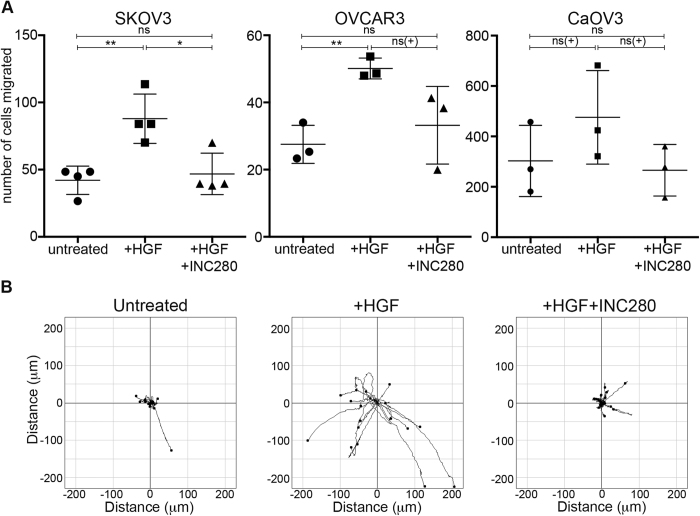
INC280 inhibits HGF-stimulated chemotactic and random migration. Boyden chamber assays were used to assess chemotactic migration toward serum in 3 cell lines (**A**). Data represents the mean from 3 separate experiments ± standard deviation. * p < 0.05, ** p < 0.005, ns(+) indicates not significant when 3 experiments are combined, although each individual assay was significant. ns, not statistically significant. Data from individual assays using CaOV3 cells has been included as Supplementary Figure 7. **B**) Migration plots of individual SKOV3 cells over 26 hrs on cell-derived matrix. Data is from single representative experiment (n = 3).

Although ovarian cancer doesn’t spread by the traditional intravasation/extravasation methods, motility remains a key aspect in metastasis. To examine the ability of INC280 to alter HGF-stimulated cell migration, we used two different assays to assess both chemotactic-induced migration (Boyden chambers), and random migration (i.e. non-chemotactic; on cell-derived matrices). As expected, addition of HGF significantly increased the number of cells migrating towards serum in the Boyden chamber assays (Fig. 3A), whereas addition of both HGF and INC280 returned migration to un-stimulated levels. Many of the signalling pathways identified as affected by HGF have also been shown to depend on cell-matrix interactions, and change in cells exposed to 3D or extra-cellular matrix^26^. While the peritoneal surface is itself 2-dimensional, we used cell-derived matrices (CDMs) to more closely recapitulate the cell-matrix interactions in the *in vivo* environment^26^, and examine the random migration of ovarian cancer cells. While OVCAR3 and CaOV3 cells were not sufficiently motile under the conditions tested, SKOV3 cells significantly extended their path lengths over a period of 26 hours with the addition of HGF (Fig. 3B, and Supplementary Figure 2), whereas co-addition of INC280 returned path length, displacement, and persistence to that of untreated cells (Supplementary Figure 2).

### INC280 inhibits peritoneal adhesion ex vivo

**Figure 4:**
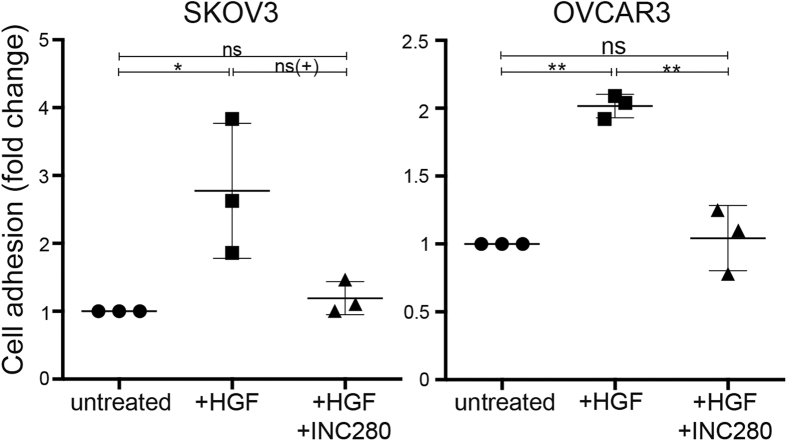
INC280 inhibits HGF-stimulated cell adhesion to peritoneum. Changes in the adhesion of SKOV3 and OVCAR3 cells to mouse peritoneum was measured in the presence of HGF and/or INC280, and is represented here as mean fold-change relative to untreated ± SD (n = 3). * p < 0.05, ** p < 0.005, ns(+) indicates not significant when 3 experiments are combined, although each individual assay was significant. ns, not statistically significant.

One of the malevolent behaviours of ovarian cancer is its adhesion to peritoneal surfaces and subsequent invasion/colonization to form widespread metastases throughout the peritoneal cavity. To address the ability of HGF and INC280 to affect the ability of ovarian cancer cells to adhere to peritoneum, we performed *ex vivo* adhesion assays using vital tissue removed from mouse peritoneum. HGF increased peritoneal adhesion of OVCAR3 and SKOV3 cells approximately 2-fold, while treatment with INC280 abrogated this effect (Fig. 4). CaOV3 cells were not included in this assay due to the fact that they invaded the peritoneum within the duration of the assay, making imaging and quantification impossible.

## Discussion

Ovarian cancer is responsible for 1 in 20 cancer-related deaths in women in the USA^1^, and the stagnation in survival rates over the past 2 decades indicate a need for improved therapeutics. One of the major factors contributing to the poor survival rates of ovarian cancer is the fact that most diagnoses are made once the disease has already metastasized throughout the peritoneal cavity^27^. It is this proclivity for disperse peritoneal/omental metastases that makes ovarian cancer difficult to treat effectively. For most tumour types, metastasis involves detachment from the tumour mass, invasion through surrounding tissue, followed by intravasation into blood or lymph vessels, extravasation into a new site, and colonization/proliferation. For ovarian cancer however, the process is different, and can be broken down into 6 main steps: 1. Cell-cell detachment, and sloughing off the primary tumour^28^,^29^. 2. Floating in peritoneal/ascites fluid, spheroid formation, and avoiding anoikis^24^,^30^,^31^. 3. Attachment of cells to mesothelium^24^,^32^,^33^. 4. Migration^34^,^35^ to favoured areas of the peritoneum or omentum (including blood vessels and milky spots^29^,^36^,^37^,^38^,^39^,^40^). 5. Invasion through mesothelium to the extracellular matrix below^33^,^35^. 6. Colonization and proliferation to form metastases^41^,^42^.

HGF and activated c-MET have been implicated in all of these steps (references given above), making the signalling pathway a valid therapeutic target. Furthermore, high expression of c-MET has been identified in 30–70% of ovarian cancer cases, and correlated with poorer prognosis^12^.

To date, a number of therapeutics blocking this pathway have been examined in phase I trials, but only one has been examined in a phase II trial specifically addressing ovarian cancer; the anti-HGF IgG_2_ humanized antibody, AMG 102^43^,^44^. The conclusion of the study was that although the therapeutic was well tolerated, it showed insufficient activity to merit further investigation as a single agent. It is possible that problems in efficacy arise from ineffective penetration of the antibody into solid tumour, the inability to completely sequester the HGF ligand, and ligand-independent activation of c-MET, all of which constitute reasons as to why small molecule inhibitors, such as INC280, should be investigated for their efficacy.

We have reported here the first investigations of the small molecule c-MET inhibitor INC280 in well characterized ovarian cancer cell line models, as well as normal human ovarian surface epithelium cells. Using the metastatic process of ovarian cancer as the basis for our experimental work, we have shown in 3 ovarian cancer cell lines that INC280 treatment reduces or abrogates pertinent cell behaviours including motility and adhesion, using HGF stimulation to mimic the presence of HGF in ascites in ovarian cancer patients^17^,^45^. INC280 treatment also abrogates HGF-induced c-MET phosphorylation in the normal epithelial cells. The sustained up-regulation of phosphorylated c-MET with HGF stimulation seen at 24 hours in SKOV3, OVCAR3, and CAOV3 cells most likely indicates additional perturbed signalling pathways which are absent in the normal cells, potentially involving mutations in Ras or alterations in splicing of CD44, both of which are proposed to perpetuate c-MET signalling^46^,^47^, and are frequently seen in ovarian cancer^48^,^49^.

The persistent inhibitory effect of INC280, is consistent with studies reported by Liu *et al*., who demonstrated that a single dose of INC280 *in vivo* resulted in 90% inhibition of c-MET phosphorylation at 7 hrs post-injection^25^. Sustained inhibition was also seen for ERK and AKT signalling downstream of c-MET, indicating the potential for this agent to be delivered once or twice daily, and maintain sufficient plasma concentrations to alter signalling.

Results regarding the ability of c-MET inhibitors to decrease cellular proliferation appears mixed, with some authors finding decreased proliferation *in vivo*^23^,^24^,^42^ and *in vitro*^22^, and others finding no changes^12^. Our initial analyses indicate that any (modest but not significant) increases in proliferation resulting from HGF stimulation are inhibited by addition of INC280. We also show no effect on proliferation with the addition of INC280 alone, unsurprising given the low levels of phosphorylated c-MET our cell lines express (Fig. 1A). Sawada and colleagues postulated that c-MET is involved in peritoneal adhesion and dissemination, but not tumour growth^12^. HGF and activation of c-MET have previously been implicated in conferring resistance to apoptosis and anoikis in ovarian cancer cells^13^,^24^,^31^. However, in our preliminary investigations, no changes were seen in the number of adhered cells, nor in PARP cleavage in attached or detached cells (data not shown), in the presence of HGF or HGF + INC280, leaving the effect of INC280 on apoptosis undetermined. However, in the original paper characterizing INC280, Liu *et al*., demonstrated that INC280 induced apoptosis in the lung and gastric cancer cell lines, H441 and SNU-5 respectively, indicating that further investigation is warranted^25^.

HGF was originally known as ‘scatter factor’, for its ability to induce an invasive phenotype in epithelial cells *in vitro*^29^,^38^,^39^. Ovarian cells migrate in a 2-dimensional setting over peritoneal surfaces. There is evidence that they preferentially colonize regions near blood vessels in the peritoneal membrane^40^ and milky spots in the omentum^36^,^37^,^50^, although little evidence exists to date as to whether cells adhere elsewhere and migrate toward these sites, or whether they attach and adhere near such sites. Regardless, the presence of high levels of HGF in ascites^51^, and its expression by peritoneal mesothelium when inflamed^32^,^33^, is likely to stimulate migration of ovarian cancer cells upon the peritoneal surface. We have addressed the effect of HGF, and subsequent inhibition of c-MET, on migration of ovarian cancer cells in both directional (chemotactic) and random migration assays, to more accurately recapitulate the environment and proteins encountered in an *in vivo* setting. Our results further support INC280 as an effective treatment in ovarian cancer, by preventing the migration of cells toward favoured sites of colonization.

The final aspect of ovarian cancer metastasis addressed here is the ability of INC280 to inhibit HGF-induced adhesion to peritoneum, one of the most important, and defining, features of ovarian cancer. It has previously been demonstrated that cells transduced with a siRNA directed against c-MET decreases adhesion to human peritoneum *ex vivo*, and mouse peritoneum *in vivo*^12^, and that cells treated with Foretinib (c-MET and VEGFR inhibitor) have decreased adhesion to a model of peritoneum using primary mesothelial cells^24^, although none of these experiments were performed in the presence of HGF. We have utilized an *ex vivo* assay to demonstrate that in the presence of HGF, INC280 acts to decrease cellular peritoneal adhesion, and thus may be useful in decreasing metastasis in ovarian cancer post-surgery. We are currently investigating whether animal models of metastatic ovarian cancer exhibit high levels of HGF and/or c-MET phosphorylation, and planning pre-clinical studies using INC280.

We have provided evidence of the potential efficacy of the c-MET inhibitor INC280 to inhibit many of the metastatic behaviours exhibited by ovarian cancer cells *in vitro* and *ex vivo*. Rather than using a ‘one size fits all’ approach however, we propose first that a biomarker such as high HGF concentrations in ascites, or high c-MET/phosphorylated c-MET in tumour samples, be used to select patients for therapy with small molecule c-MET inhibitors. Furthermore, small molecule c-MET inhibitors, including INC280, should be tested in combination with existing therapies in models of ovarian cancer post-debulking surgery, to address their usefulness in preventing survival, adhesion, and migration of cells remaining within the ascites fluid, and potentially preventing growth of micro-metastases.

## Methods and Materials

### Reagents

INC280 was obtained from Novartis and used at a final concentration of 12 nM, previously established to decrease c-MET phosphorylation by approximately 80% (Supplementary Figure 3). HGF (Symansis) was used at a final concentration of 40 ng/mL. Antibodies against total c-MET (4560), phospho-c-MET (Y1234/1235; 3077), total ERK1/2 (9102), phospho-ERK1/2 (Y202/204; 9101), total AKT (9272), phospho-AKT (S473; 4058) were from Cell Signaling Technology. β-actin (5541, Sigma) was used as a loading control.

### Cells and media

The human epithelial ovarian cancer cell lines OVCAR3 and SKOV3 were cultured in RPMI 1640, CaOV3 cells in DMEM, and normal human ovarian surface epithelial cell line HOSE 6.3 cells in Medium 199:MCDB105 1:1. All media contained 10% FCS, and all lines were purchased from the American Type Culture Collection, and their identity confirmed by short tandem repeat analysis.

### Western blotting

For the detection of activated signalling pathways, cells were seeded into 6 well plates for 24 hours prior to treatment. For samples treated with INC280, cells were pre-treated with INC280 for 10 mins, prior to addition of fresh media containing HGF/INC280 for the indicated time periods. Cells were lysed, and proteins separated by SDS-PAGE.

### Proliferation

Confluence was measured as a surrogate marker for proliferation, using INCUCYTE^TM^ live-cell imaging system (Essen Bioscience). Cells were seeded into a 96 well plate (per well: 2500, CaOV3; 5000, OVCAR3; 1500, SKOV3; 2000, HOSE 6.3), allowed to adhere overnight, serum-starved the following night, and then treated with HGF and/or INC280. Media was replenished every 48 hrs. Data was normalized to confluence levels post serum-starvation, and the end-point for each cell type was defined by any well reaching 90% confluence. Analysis was performed using GraphPad Prism.

### Migration

Boyden chamber assays were performed using 8 μm transwells. Cells were serum-starved overnight, and seeded into transwells in 24 well plates (per well: 3.75 × 10^4^, CaOV3; 1.43 × 10^5^, OVCAR3; and 3.58 × 10^5^, SKOV3), in the presence of HGF/INC280 as indicated. Serum-containing media with the same HGF/INC280 treatment was used in the wells. Cells were allowed to migrate over 6 hrs, then fixed and stained using DiffQuik. Cell counts were obtained, and statistical analysis performed using GraphPad Prism.

For random migration assays, cell-derived matrix (CDM) was generated as described previously^52^ in 6 well plates. Cells were seeded at 5 × 10^4^/well in media containing HGF/INC280, and left to adhere for 10 hrs, before beginning monitoring under bright field time-lapse microscopy, with images obtained every 15 mins, for approximately 24 hrs. Cell migration was analysed using the MTrackJ ImageJ plugin, tracking only cells that did not divide during the relevant period. Data analysis was performed in GraphPad Prism.

### *Ex vivo* peritoneal adhesion assays

*Ex vivo* peritoneal adhesion assays were performed as previously described^53^. Briefly, peritoneal tissue was excised form 10–12 wk. female Balb/c mice, and placed into serum-free media. Syto9-labelled cells (1 × 10^5^ were added to 96 well plates, along with HGF and or INC280 at final concentrations of 40 ng/mL and 12 nM respectively. The tissue was laid over the top of the wells, mesothelial side down, covered by a glass coverslip, and the inverted plate then incubated for 3 hrs at 37 °C. The peritoneal tissue was then washed with serum-free medium, and attached cells observed and imaged. Image J was used to count 6–9 fields/well. Animal experimentation described within this study was carried out in accordance with the guidelines approved by the Garvan Institute and St Vincent’s Hospital Animal Ethics Committee.

### Statistics

Statistical analyses were performed using GraphPad Prism, using two-tailed, un-paired t-tests, assuming equal standard deviations, with significance defined as p < 0.05. Data points are presented as mean ± standard deviation (SD).

## Additional Information

**How to cite this article**: Moran-Jones, K. *et al*. INC280, an orally available small molecule inhibitor of c-MET, reduces migration and adhesion in ovarian cancer cell models. *Sci. Rep*. **5**, 11749; doi: 10.1038/srep11749 (2015).

## Supplementary Material

Supplementary Information
