# Supplementary material for: INC280, an orally available small molecule inhibitor of c-MET, reduces migration and adhesion in ovarian cancer cell models
Source: Sci Rep. 2015 Jul 3;5:11749. doi: 10.1038/srep11749 (PMC5155610; doi:10.1038/srep11749)
Supplement: Supplementary Information [file srep11749-s1.pdf]

INC280, an orally available small molecule inhibitor of c-MET, reduces migration and adhesion in ovarian cancer cell models.

Kim Moran-Jones,
Laura M. Brown,
Goli Samimi.

Supplementary Figure 1

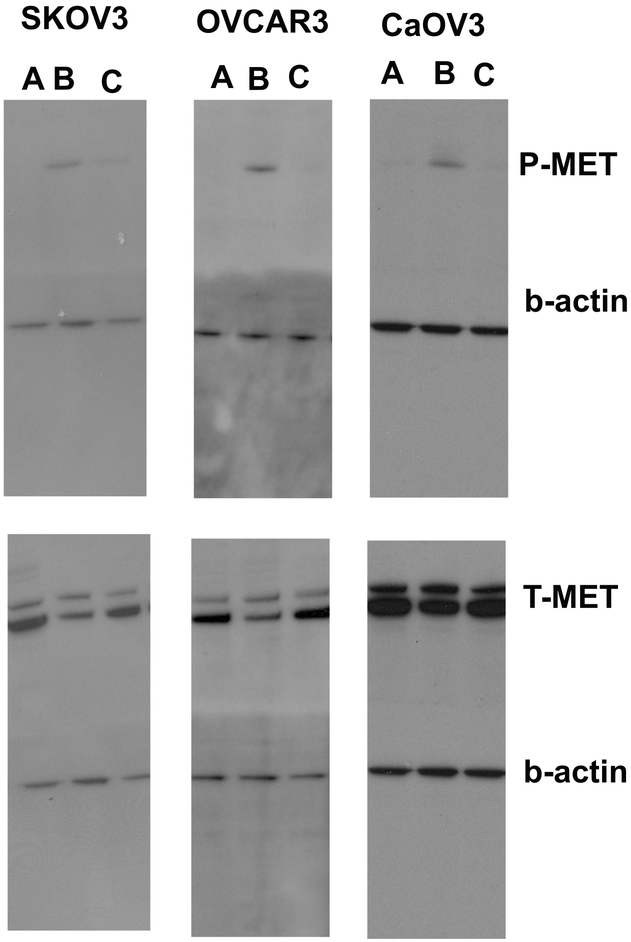

A: untreated
B: HGF
C: HGF+INC280

Cells were treated for 48 hous with the indicated treatments, before being lysed, and blotted for phsopho- and total c-MET. b-actin was used as a loading control.

Untreated

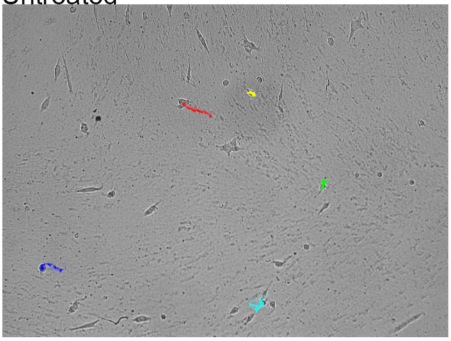

+HGF

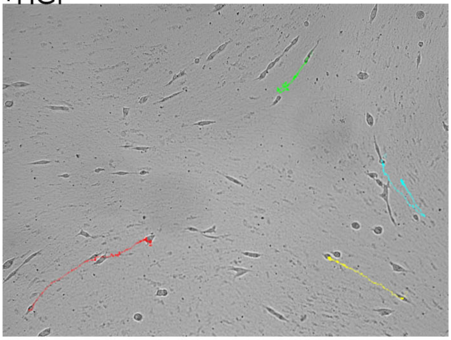

+HGF+INC280

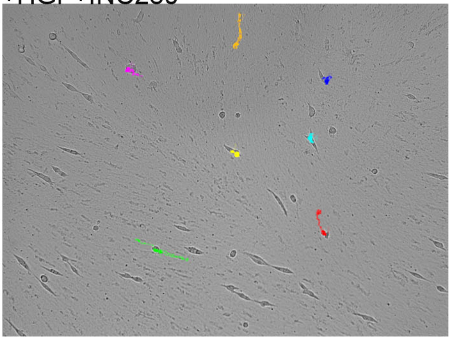

Supplementary Figure 2

SKOV3 cells were tracked during their migration over 24 hours on cell-derived matrices. Representative tracks from each condition are shown to the left, where cells are located in their end positions. Graphs of displacement, pathlength, and persistence are located below, and are plotted as means of 3 independent experiments.

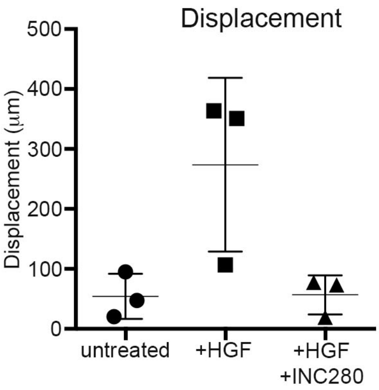

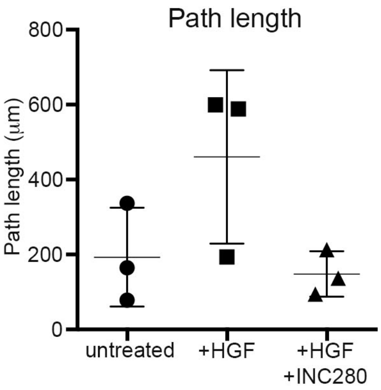

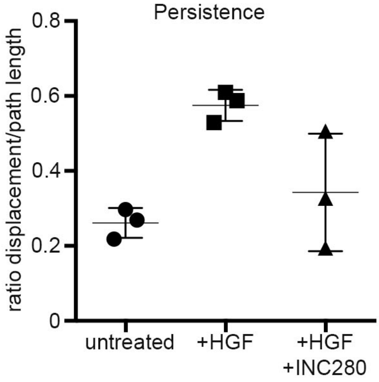

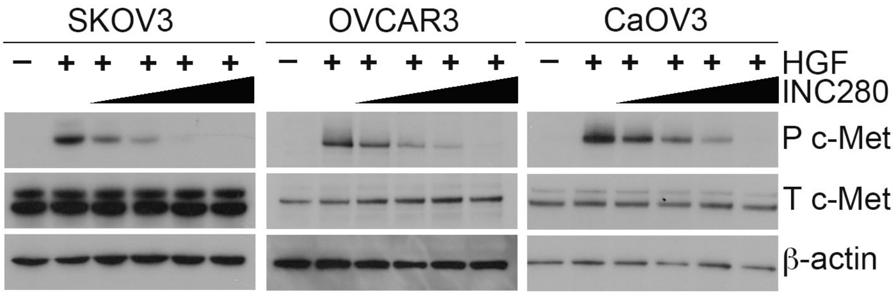

SKOV3, OVCAR3, and CaOV3 cells were treated with HGF and INC280 (3, 6, 12, or 30 nM final concentration). Westerns were performed, and blotted for phospho- and total c-Met. b-actin was used as a loading control.

Supplementary Figure 3

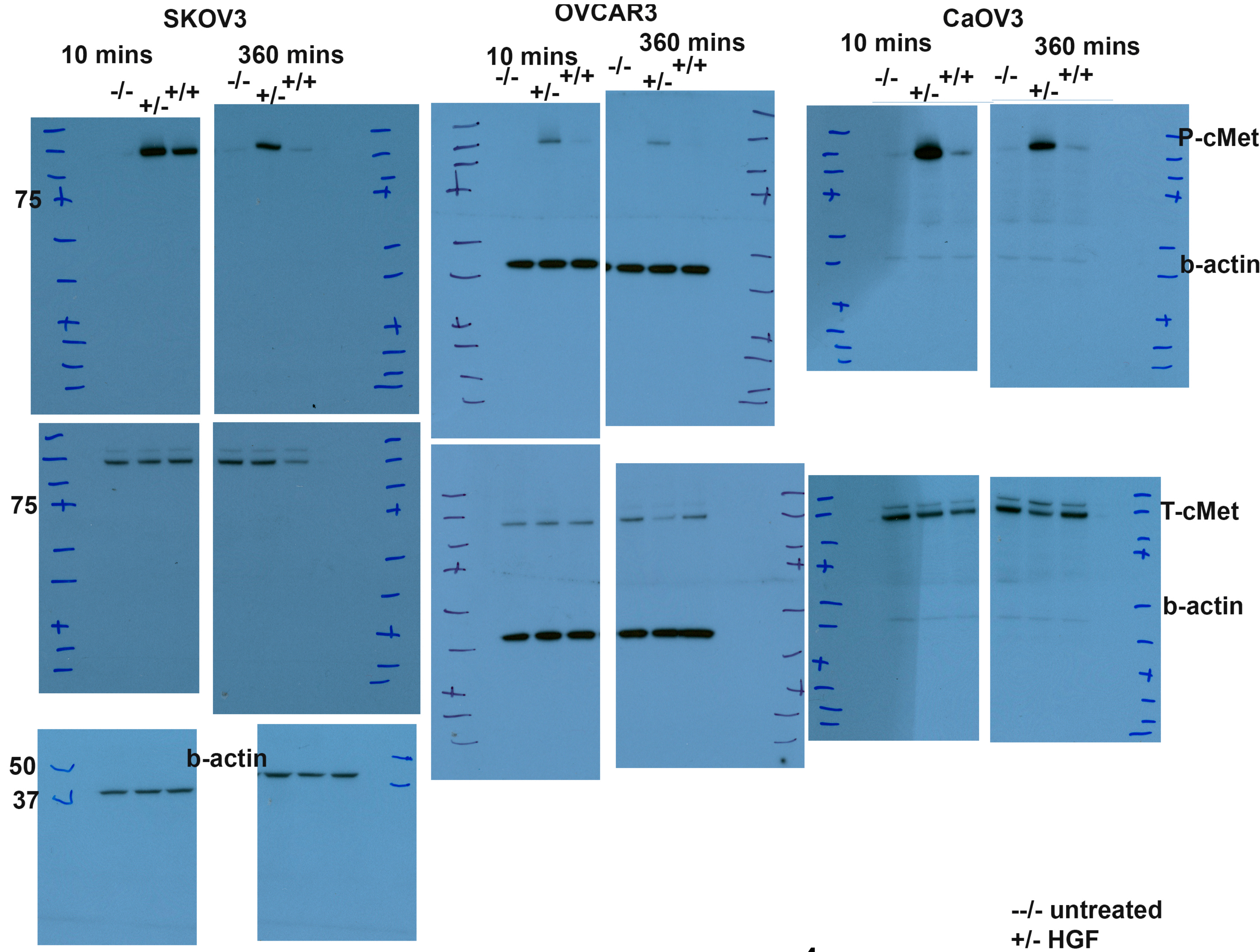

Supplementary Figure 4

--/- untreated
+/- HGF
+/+ HGF & INC280

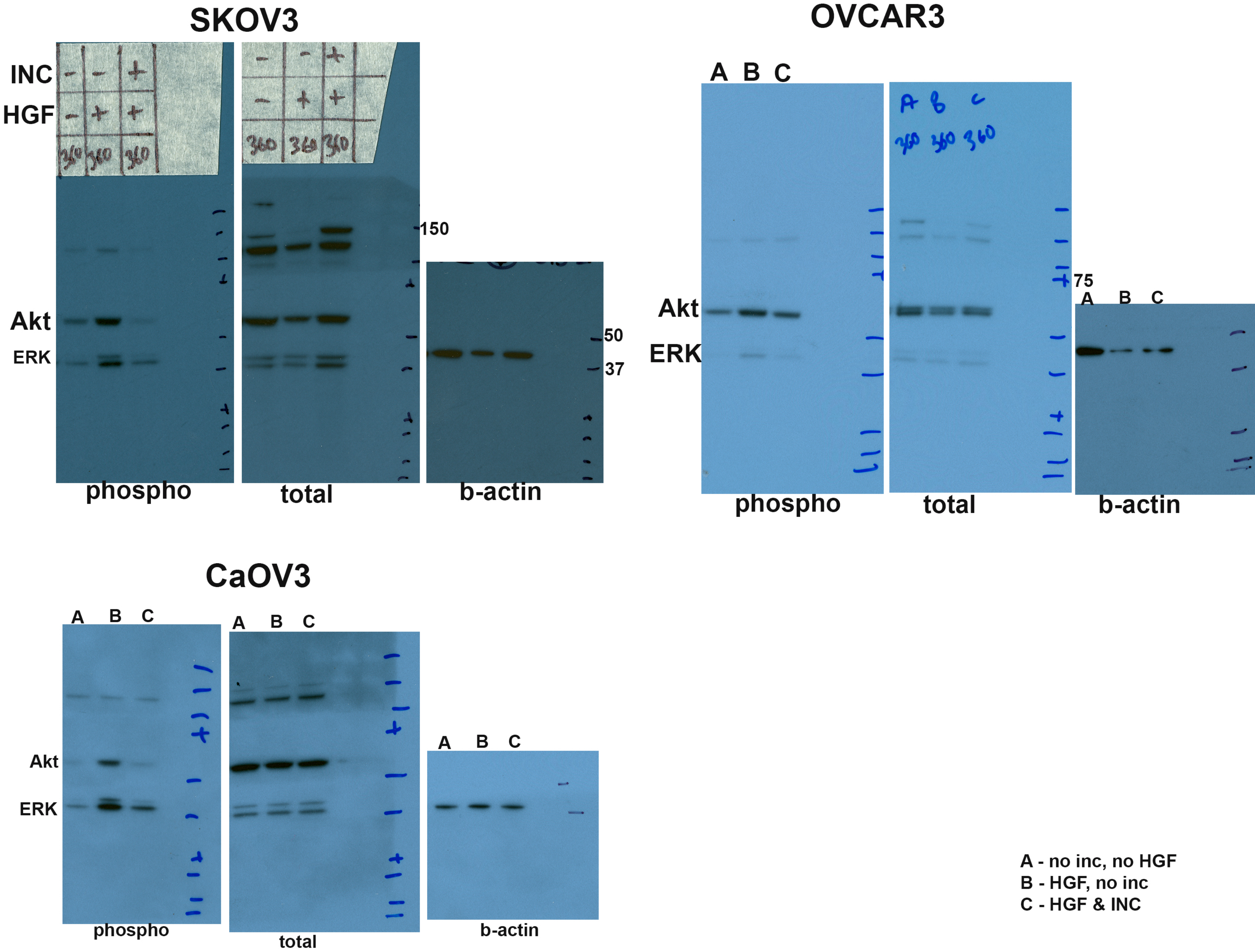

A - no inc, no HGF
B - HGF, no inc
C - HGF & INC

Supplementary Figure 5

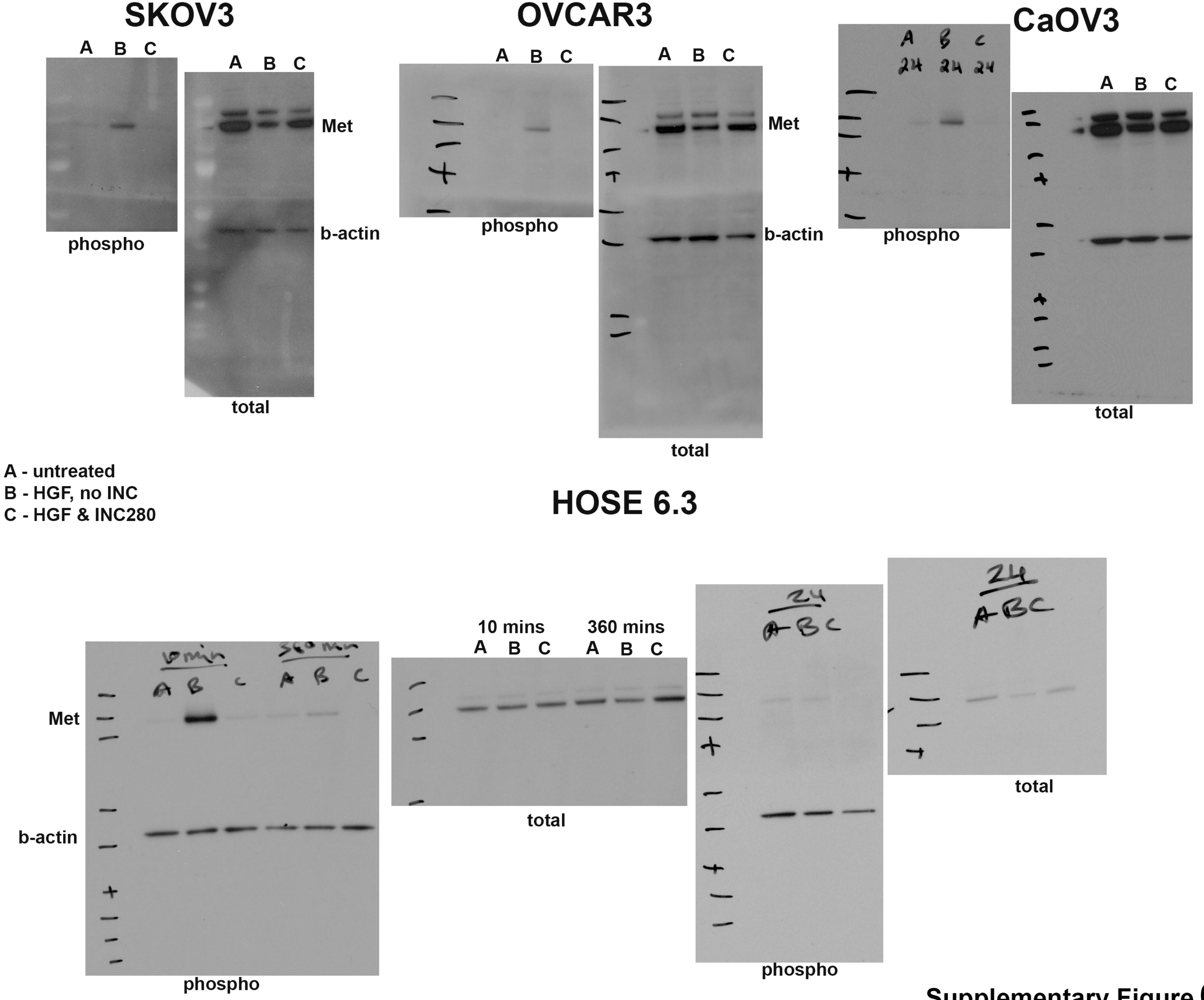

Supplementary Figure 6

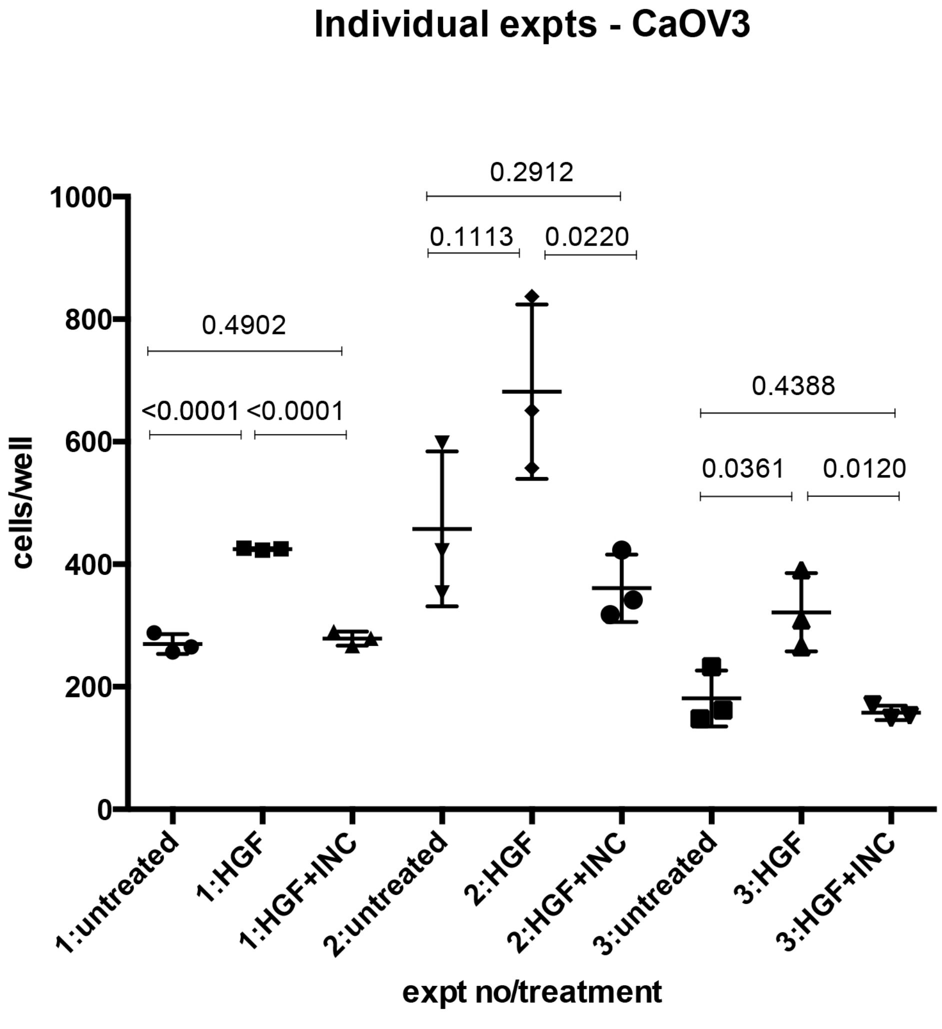

Supplementary Figure 7
p-values for individual experiments are indicated
